# Polymorphims of innate immunity genes influence disease progression in HIV-1 infected children

**DOI:** 10.1186/1742-4690-9-S1-P14

**Published:** 2012-05-25

**Authors:** Riccardo Freguja, Ketty Gianesin, Marisa Zanchetta, Francesco Carmona, Sandro Malacrida, Osvalda Rampon, Carlo Giaquinto, Anita De Rossi

**Affiliations:** 1 Researcher at University of Padua, Padua, Italy

## Introduction

Toll-Like Receptors (TLRs) and Defensins play a crucial role in host's innate immune response. Genetic variations in Defensins and TLRs may affect host-virus interactions and impact HIV1 disease progression, particularly in infants who acquire immune infection when adaptive immune response is still under development.

## Methods

The study was performed in 95 perinatally HIV1 infected children followed since birth. The endpoint was the onset of disease (stage C) or initiation of highly active antiretroviral therapy. The median (interquartile) followup from birth to endpoint was 87 (46-134) months. Single nucleotide polymorphisms (SNPs) on Beta-defensin1 (DEFB1 -44C>G;-52G>A), and TLR9 (1174G>A;1635A>G) genes were identified with TaqMan allelic discrimination assay. The probability of acquiring disease was calculated with Kaplan-Meier method. Hazard ratios and their 95% confidence interval (95% CI) based on the Cox proportional hazards model were estimated to test the association between genotypes, haplotypes and risk of stage C.

## Results

TLR9 1635AG genotype was associated with rapid disease progression with both KaplanMeier (p=0.008) and Cox analysis (p=0.009), while DEFB1 -44CG genotype was associated with slower disease progression with both KaplanMeier (p=0.020) and Cox analysis (p=0.024). Notably, TLR9 [G;G] haplotype, previously associated with a higher risk of mother-to-child transmission of HIV1 (MTCT), was also found to be associated with rapid disease progression (p=0.033). In addition, DEFB1 [G;G] haplotype, found to be protective against MTCT, was associated (p=0.016) with a better clinical outcome in HIV1 infected children.

## Conclusions

Overall, these findings support the role of innate immunity in pediatric HIV1 pathogenesis. Specific SNPs in DEFB1 and TLR9 genes may affect the functional ability of their encoded proteins to modulate innate immunity, thus contributing to the variability of clinical outcome in HIV1 infected children.

